# Time Interval between Self-Detection of Symptoms to Treatment of Breast Cancer 

**DOI:** 10.31557/APJCP.2020.21.1.169

**Published:** 2020

**Authors:** Shivaraj Nallur Somanna, Murthy Nandagudi Srinivasa, Ramesh Cheluvarayaswamy, Nea Malila

**Affiliations:** 1 *Department of Community Medicine, Ramaiah Medical College, *; 2 *Department of Epidemiology and Biostatistics, Kidwai Memorial Institute of Oncology, Bangalore, India, *; 3 *Finnish Cancer Registry, Finland. *

**Keywords:** Breast cancer, time interval, breast cancer symptoms, seeking cancer care

## Abstract

**Background:**

In India breast cancer is the number one cancer among females with an incidence rate of 25.8 per 100,000 women and mortality of 12.7 per 100,000 women. India continues to have a low 5-year survival rate of breast cancer with only 66.1% as compared to 90% in developed countries. The major reason for low survival is that patients are diagnosed with cancer at high stage. The present study attempts to delineate the time interval between self-detection of breast cancer symptoms and seeking care and to find the main reasons for delay in seeking care.

**Methods:**

A cross sectional study was undertaken from October 2016 to March 2017 in a population based cancer registry (PBCR) and hospital based cancer registry (HBCR) located in south of India. Histologically confirmed breast cancer patients (N=181) were interviewed at hospital using a pre-tested semi structured questionnaire.

**Results:**

The median time interval between the self-detection of breast cancer symptoms and first contact with general physician was 60 [IQR 30-180] days. The median time to diagnosis from the first contact was 30 [IQR 10 - 60] days and the overall median time span from self-detection of symptoms to treatment was 150 [IQR 95-265] days. The major reason given for not seeking medical care in time was lack of awareness in identifying the cancer symptoms both among patients and primary care providers.

**Conclusion:**

There was considerable delay from self-detection of symptoms to cancer specific primary treatment of breast cancer. We found lack of awareness among patients as well as in primary care providers to be the major concern for delay. Awareness among the target population and health care professionals would have to be improved for early diagnostics and access to care.

## Introduction

Worldwide there were 2.1 million newly diagnosed female breast cancers in 2018 and in most of the countries it was the leading cancer among women as well as the leading cause of death globally (“Global cancer statistics 2018: GLOBOCAN estimates of incidence and mortality worldwide for 36 cancers in 185 countries - Bray - 2018 - CA: A Cancer Journal for Clinicians - Wiley Online Library,” n.d.). Incidence of breast cancer is rising rapidly in economic emerging countries like Asia and Africa. It is expected that the incidence of breast cancer would be 1.7 million cases in 2020 with 70% mortality occurring in the developing world (Rivera-Franco and Leon-Rodriguez, 2018). The possible reason for such high mortality could be limited resources for prevention, diagnosis and treatment of cancer.

In India, breast cancer is the number one cancer among females with an incidence rate of 25.8 per 100,000 women and mortality of 12.7 per 100,000 women, according to health ministry (Malvia et al., 2017). India continues to have a low 5-year survival rate of breast cancer with only 66.1% as compared to 90% in developed countries for women diagnosed with disease between 2010 and 2014 (Allemani et al., 2018). The major reason for low survival is that majority of the patients seek care at late stages. Also affordability, access to care and awareness are key barriers in diagnosis and treatment in India (Tripathi et al., 2014). 

Although many studies have identified the time between self- detection of cancer symptoms to first consultation and its barriers, there are few studies which have been studied the time interval between cancer symptoms to first consultation, to referral by general practitioner, to tertiary care, to definite diagnosis and treatment, and the reasons for any delay (Gangane et al., 2015; Thakur et al., 2015; Tripathi et al., 2014).

The present study aims to delineate the time interval between self-detection of breast cancer symptoms and seeking care and to find out what were the reasons for any possible delay.

## Materials and Methods

A cross sectional study was undertaken from October 2016 to March 2017. Breast cancer cases with histological confirmation were recruited from a cancer registry located at Bengaluru, India, within a tertiary care hospital with state of art facilities for diagnosis, treatment and research. This hospital has both a population based cancer registry (PBCR) and a hospital based cancer registry (HBCR) which is offering services to the patients from all over Karnataka state. Majority of the patients seek cancer care from this hospital as it is a regional cancer center. Breast cancer patients referred to palliative care for end of life support were excluded from the study. 

The sample size was estimated to look at the sufficiency of the estimated parameter with the desired precision based on the study conducted by Moodley et al., (2018) where the overall median time from the symptom discovery to initiation of treatment was 110 [IQR 67-178] days. Considering a 5% alpha error and a relative precision of 7.3%, the study required a minimum sample of 180 subjects. In order to get this samples, based on hospital records, it was noted that six months’ time period was sufficient to achieve the required sample size. 

*Data collection*

**Figure 1:**
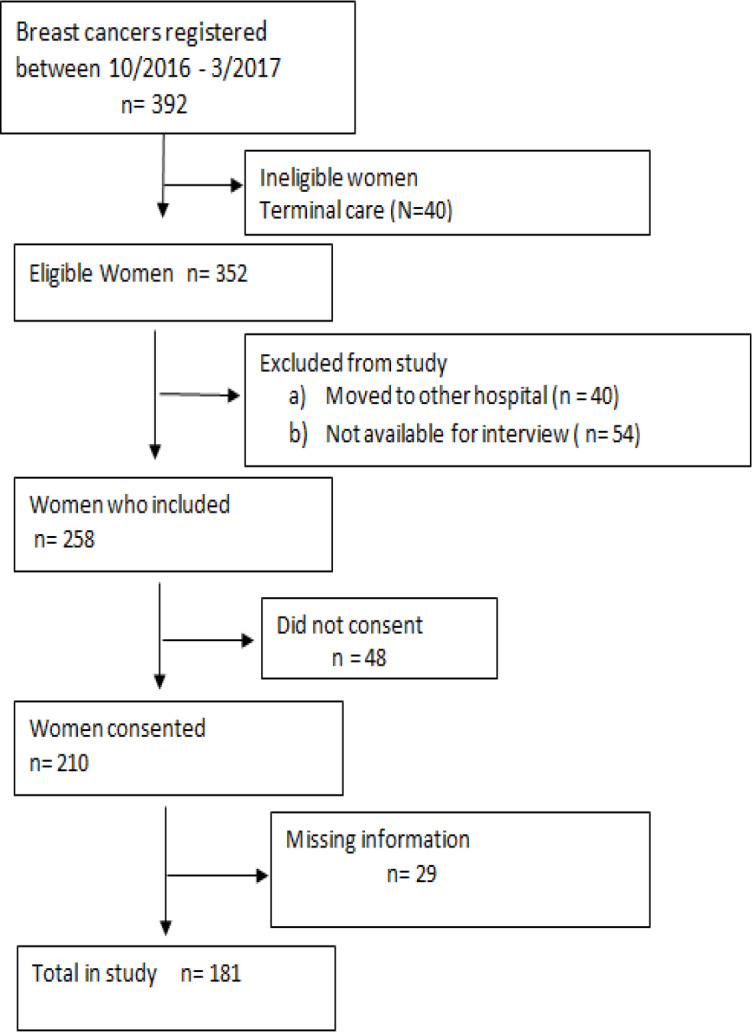
Recruitment of Participants for the Study

We identified 392 breast cancer patients diagnosed during the period from October 2016 to March 2017. Of these, 40 were ineligible due to refer to terminal/palliative care. Among 352 eligible women, 84 were excluded from the study since the patient moved to another hospital or not available for interview and 48 did not consent for the study. Of 210 patients who were consented, 29 patient’s information were incomplete in the record. Finally, 181 patients were enrolled for the present study (Figure 1). Tumors of stage I and II were categorized as early stage, III and IV as late stage of breast cancer.

A semi-structured questionnaire was developed with expert inputs from clinical oncologists and epidemiologists and piloted for assessing appropriateness and face validity. After obtaining an informed consent, patients were interviewed using this pre-tested questionnaire to collect base line information as well as key components of the temporal events from self-detection of breast cancer symptoms to cancer specific primary treatment. Clinical and pathological details of the disease and socio-demographic details were extracted from the hospital records. 

The time interval was measured in days reported by the patients and from review of hospital case sheets for the various components as indicated below (Somanna et al., 2018).

a) Time interval between self-detection of symptoms and the first presentation at general practitioner (T_1_).

b) Time interval from referral by the general practitioner to attending tertiary care center (T_2_). 

c) Time interval from first consultation at tertiary care for diagnosis to definite diagnosis date (T_3_).

d) Time interval from definite diagnosis to initiation of primary treatment (T_4_). 

e) Total time elapsed from cancer symptoms to initiation of primary treatment (T_5_).

Presently there is no specific guidelines suggesting the optimal time to the initiation of breast cancer treatment. Studies have shown that delaying more than 90 days for treatment was associated with 53% increase in risk of death (Chavez-MacGregor et al., 2016). A time interval of 90 days and more for initiation of primary treatment was considered to be as delay in the present study. 

*Statistical methods*

Data on time interval in days was described with median and inter quartile range as data was not normally distributed, by Kolmogrov-Smirnov and Shapiro test. Chi Square test of significance for proportions was used to assess the association of socio-demographic factors on the delay in presentation at hospital. Spearman rank correlation co-efficient (ρ ) was used to test the relationship between the various time events. A P-value of < 0.05 was considered statistically significant. Data was analyzed using SPSS Inc. Released 2009. PASW Statistics for Windows, Version 18.0. Chicago.

Ethical clearances were obtained from the Hospital institutional ethical committee Kidwai Memorial Institute of Oncology, Bangalore, India.

## Results

In all, 181 breast cancer patients were interviewed with complete information available. The median age at diagnosis was 49 [IQR 41-56] years with the age range of 20 to 83 years. Of the patients, 73 (40.3%) were below 45 years of age, 114 (63%) were not literate, 108 (59.7%) from rural areas, and 162 (89.5%) financially dependent for livelihood on the family. Only 5 women (2.8%) had any health insurance policy. Also, the majority of cancer patients, 119 (65.8%), were in late stage at diagnosis (Table 1). Among 352 eligible women 171 could not be included in the study. Their median age was 51 (43 – 62) years, 69% were not literate and 62.4% were at late stage at diagnosis. The differences in the baseline characteristic between included and not included subjects were statistically not significant (Age: P = 0.43, Literacy status: P = 0.68, Stage: P=0.70).

The median time interval between the self-detection of breast cancer symptoms and first contact with general physician (T_1_) was 60 [IQR 30-180] days, while 70 patients (38.7%) had a time lag of more than 90 days. The median time taken for reaching diagnosis after the first contact (T_2_) was 30 [IQR 10 - 60] days, and for 116 patients (64.1%) this took more than 15 days. The overall IQR was [95-265] days. Of the patients, 140 (77.3%) had primary treatment initiation after 90 days from symptoms (Table 2). Women who delayed with their first contact (T_1_) had delayed to report at tertiary care (T_2_) after referral and their relationship was found to be statistically significant (ρ = 0.2, P = 0.03). However the relationship between the time interval for first contact (T_1_) and definite primary treatment (T_4_) (ρ= 0.140, P= 0.06) was not statistically significant (Table 3). Of all the factors associated with delay in seeking medical care, literacy status was the only factor which had a statistically significantly association (P = 0.029) (Table 4). 

While studying the factors that led to seek for medical care, majority of women 150 (82.9%) had given a lump in the breast as the reason. The other common reasons were pain in the breast in 38(21.0%) women, swelling in 21 (11.6%) women, and nipple discharge/bleeding in 10 (5.5%) women. 

The reasons leading to late seeking for medical care are given in Table 5. The majority 177 (97.7%) of patients had a delay of more than one week in seeking medical care. The major reason for not seeking medical care was lack of awareness in identifying the breast cancer symptoms in 159 (89.3%) women, followed by 80 (44.9%) patients assuming that the symptom would resolve by itself. The other common reasons were absence of pain, changes in the body attributed to common illness and vague symptoms. We observed that the most common reason for delay in getting a definite diagnosis at tertiary care hospitals was due to visits to multiple medical practitioners who did not suspect cancer. The delay in seeking for treatment after diagnosis at tertiary care was due to fear of treatment in 58 (44.3%) women, financial dependence on the family, disfiguring of the body, stigma attached with the disease, and the long treatment procedure.

**Table 1:** Distribution of Socio-Demographic, Clinical and Other Variables among Study Subjects

Variable	Frequency (%)
Age (in years)	
< 45	73 (40.3)
45 – 60	74 (40.9)
>60	34 (18.8)
Literacy status	
Not literate	114 (63.0)
Literate	67 (37.0)
Residence Area	
Rural	108 (59.7)
Urban	73 (40.3)
Financially Dependent for livelihood	
Yes	162 (89.5)
No	19 (10.5)
Family history of Breast cancer	
Yes	12 (6.6)
No	169 (93.4)
Any Health Insurance	
Yes	8 (4.4)
No	173 (95.6)
Knowledge regarding cancer cure by early seeking for care	
Yes	13 (7.2)
No	168 (92.8)
TNM Staging of the disease	
Early stage (I + II)	62 (34.2)
Late stage (III + IV)	119 (65.8)

**Table 2:** Distribution of Time Interval between Self-Detection of Cancer Symptoms to Treatment

Time Span between (N = 181)	Duration (in days)		
	Median [IQR]	Days	n (%)
Self-detection of symptoms to first presentation for medical care(T_1_)	60 [30, 180]	≤ 90	111 (61.3)
		> 90	70 (38.7)
First contact to presentation at tertiary care (T_2_)	30 [10, 60]	≤ 15	65 (35.9)
		> 15	116 (64.1)
Initial Presentation at tertiary care to definite diagnosis (T_3_)	9 [5, 15]	≤ 7	84 (46.4)
		> 7	97 (53.6)
Definite diagnosis to Initiation of primary treatment (T_4_)	16 [11, 28]	≤ 7	22 (12.2)
		> 7	159 (87.8)
Total time from onset of symptom to initiation of primary treatment (T_5_)	150 [95, 265]	≤ 90	41 (22.7)
		> 90	140 (77.3)

**Table 3:** Correlation Co-Efficient between Different Time Intervals of Seeking Cancer Care

Time Interval (N = 181)	Correlation co-efficient (ρ)	P - value
T_1_ and T_2_	0.2	0.03
T_2_ and T_3_	0.005	0.95
T_3_ and T_4_	0.14	0.061

Time interval between self-detection of symptoms and the first presentation at general practitioner (T_1_). Time interval from referral by the general practitioner to attending tertiary care center (T_2_). Time interval from first consultation at tertiary care for diagnosis to definite diagnosis date (T_3_). Time interval from definite diagnosis to initiation of primary treatment (T_4_). Total time elapsed from cancer symptoms to initiation of primary treatment (T_5_).

**Table 4:** Association of Socio-Demographic and Other Factors with Delay in Seeking Medical Care

Characteristic (N = 181)	Delay in seeking medical care		P - value
Age (in years)	≥ 90 days	< 90 days	
< 45	32 (43.8)	41 (56.2)	0.346
45 – 60	24 (32.4)	50 (67.6)	
>60	14 (41.1)	20 (58.9)	
Literacy Status			
Not literate	51 (44.7)	63 (55.3)	0.029
Literate	19 (28.4)	48 (71.6)	
Residence area			
Rural	40 (37.0)	68 (63.0)	0.582
Urban	30 (41.1)	43 (58.9)	
Financially dependent for livelihood			
Yes	62 (38.3)	100 (61.7)	0.745
No	8 (42.1)	11 (57.9	
Family history of Disease			
Yes	4 (33.3)	8 (66.7)	0.694
No	66 (39.1)	103 (60.9)	
Any health Insurance			
Yes	2 (25)	6 (75)	0.417
No	68 (39.3)	105 (60.7)	
Distance of any health center (in km)			
≤2	52 (38.5)	83 (61.5)	0.941
> 2	18 (39.1)	28 (60.9)	

**Table 5:** Distribution of Perceived Reasons for Delay in Not Seeking Medical Care at Various Stages

Delay Stage	Perceived Reasons	Frequency (%)
Self-detection of symptoms to first contact (N = 178)*	Lack of awareness of cancer symptoms	159 (89.3)
	Belief that cancer symptom would resolve by itself	80 (44.9)
	Absence of pain	71 (39.9)
	Changes in the body attributed to common illness	47 (26.4)
	Vague symptoms	43 (24.2)
	Shame associated with Disease	43 (24.2)
	Fear of Surgery	19 (10.7)
	Financial problems	19 (10.7)
	Others	10 (5.6)
Presenting at tertiary care for Diagnosis after first contact (N= 179)*	Visited multiple medical practitioner before presenting at tertiary care (primary care providers)	71 (39.7)
	Affordability	61 (34.1)
	Not suspected for cancer at first contact	50 (27.9)
	Distance of tertiary care center	42 (23.5)
	Nobody to accompany	29 (16.2)
Treatment after diagnosis (N = 131)#	Fear of treatment	58 (44.3)
	Dependent on the family	51 (38.9)
	Treatment would disfigure the body	51 (38.9)
	Affordability	46 (35.1)
	Long treatment procedure	45 (34.4)
	Treatment Not comfortable	41 (31.3)
	Stigma	31 (23.7)
	Distance of Hospital	11 (8.4)
	Loss of wage	15 (11.5)

*, Responses was elicited from those who had not sought specific care for more than one week from the onset of symptoms; #, Responses was elicited from those who had not sought treatment on specified time by doctor.

## Discussion

This study was done in a population and hospital based cancer registry where the majority of breast cancer patients seek for care. Our study observed that the median time interval between the self-detection of breast cancer symptoms and first contact with a general physician was 60 [IQR 30-180] days. The median time span to treatment from self-detection of symptoms was 150 [IQR 95-265] days. 

It has been reported from the Bangalore cancer registry that breast cancer is more common in the younger age group and 53.2% of all women suffering from breast cancer were below 50 years of age compared to developed country USA (Breast cancer India, n.d.). In the present study we observed that cases diagnosed below age 45 years accounted for 40.3%. Other studies from India have indicated that 38.4% of breast cancers were diagnosed in the age group of ≤ 40 years (Pakseresht et al., 2014). Similarly, in a study done by (Chandra Roy et al., 2015) in Bangaldesh the average age of breast cancer patients was 49.16 (SD ± 11.79) years. In US, (Street, n.d.2017) report shows the average age of breast cancer diagnosis is 62 years. This differences in the age at onset of breast cancer cases may be due to differences in the life expectancies of India and US. Several studies have reported that the delay in seeking care is often associated with low socio economic status (Kumar et al., 2017; Rath et al., 2018). Similar findings are seen in the present study. In low resource settings where the level of education is often low which results in poor economic status leading to altered priorities of basic sustenance rather than seeking health care.

The delay in seeking for breast cancer care affect patient’s survival. Our current study addressed the time interval between self-detection of symptoms to treatment of breast cancer. We observed that for close to 40% of patients it had taken more than 3 months for their first contact with a general physician (T1) after self-detection of symptoms. A study by Gangane et al., (2015) at a tertiary care center observed that 49% of patients had delayed for initial presentation for medical care. In a study at National university Hospital, Singapore and university of Malaya Medical Centre in Kuala Lumpur by Lim et al. also found that 46% patients had an interval of 3 months and over between symptom discovery and first presentation for medical care (Lim et al., 2015). Our observations were similar to these other study findings.

The current study observed that the women who had delay in the first contact (T1) also had significant delay in reporting at tertiary care centre (T2) after referral. As noted from the perceptions for the delay, majority i.e 39% of women visited multiple practitioners before presenting at tertiary care. Length of diagnosis has been found to be associated with number of hospitals visited (Shieh et al., 2014). Study by Moodley et al., (2018) also showed that 40% of women had visited 4 or more health care visits from the time of first symptom discovery and definitive breast cancer diagnosis. Other reasons for the delay in seeking diagnosis and treatment was affordability and not suspected for the breast cancer. 

Our study indicated that 66% of the patients were diagnosed in late stage (III + IV) of breast cancer. A study from India 66.9% were diagnosed in late stage which was similar to our findings (Pakseresht et al., 2014). In a similar study from a hospital based cancer registry 54% were diagnosed in late stage (Sathwara et al., 2017). Another study based on a tertiary care center hospital observed 58% in late stage (Thakur et al., 2015). Our findings indicate slightly higher percentage of late stage disease possibly due to majority of patients belong to lower economic status. This referral center comes under the government of Karnataka where various government scheme are provided for the treatment at free of cost who do not have health insurance and belongs lower economic status.

In our study, uni-variate analyses between patient’s socio-demographic characteristics and delay in seeking medical care indicated a statistically significant association for education status. A study by Partridge et al., (2012) showed that young women were likely to have delay in diagnosis. In the present study, those who were literate presented for medical care early compared with those who were not literate (P =0.029). However, living in an urban area, having a family history of cancer, having health insurance coverage or being in close proximity to a health center did not translate into seeking early medical care. A study by Lopes et al., (2017) had similar findings with no difference in delay in treatment seeking between younger and older age groups, living near or far from the municipality hospital and having family history of cancer, but years of education played a role in seeking earlier care. Similarly to our study, education and financial constraints were the main reasons for more than 3 months delay in seeking care in the study by Shieh et al., (2014). 

One of the most perceived reasons for delayed care in the present study was the lack of awareness of cancer symptoms in 89% of women and in 45% the women thought that the symptom would resolve by itself. Similar findings were observed in a qualitative study done by Ilaboya et al., (2018) in Uganda where all the participants had poor knowledge or complete lack of knowledge, which was a key barrier to early detection of breast cancer. In an another study from Egypt Ismail, (2013) patients had a negative interpretation of cancer symptoms, i.e. lack of awareness about breast cancer, or they denied having breast cancer and thought the mass would resolve on its own. About 40% of the patients in our study did not perceive any pain which led to delayed medical care. Similarly in a study done by Otieno et al., (2010) a painless lump and not being aware of the disease led to delayed care. 

*Strengths and Limitation*

The strength of this study is that it has been carried out in population based cancer registry where the data collection procedure in this center involves various inbuilt quality control measures and the data from this PBCR are included in International Agency for Research on Cancer (IARC). Cases included have been diagnosed using standard clinical and histopathological criteria by experts. The information was obtained using pre-tested validated questionnaire by the investigator. Additional information pertaining to clinical evaluation and histological diagnosis were obtained from case sheet. Lower rate of non-response among eligible ensures representativeness. The social strata of the population who avail the services at the center may be different from the general population and private cancer center. In the absence of mass screening programme for detection of cancer in the country, sample in the study represent only those who avail the treatment at the center. We understand the possibility of recall bias as past history of cancer symptoms were asked.

In conclusion, the findings of this study identified delays at all stages of cancer care from the self-detection of symptoms to cancer specific primary treatment. We found lack of awareness both among the patients and among the primary care providers to be the major concern. This information will be useful for planning cancer control programs. Awareness among the target population and health care professionals would have to be improved for early diagnostics and access to care.
